# Comparison of Gene Expression Between Resistant and Susceptible Families Against VP_AHPND_ and Identification of Biomarkers Used for Resistance Evaluation in *Litopenaeus vannamei*

**DOI:** 10.3389/fgene.2021.772442

**Published:** 2021-11-26

**Authors:** Qian Zhang, Yang Yu, Zheng Luo, Jianhai Xiang, Fuhua Li

**Affiliations:** ^1^ Key Laboratory of Experimental Marine Biology, Institute of Oceanology, Chinese Academy of Sciences, Qingdao, China; ^2^ University of Chinese Academy of Sciences, Beijing, China; ^3^ Laboratory for Marine Biology and Biotechnology, Qingdao National Laboratory for Marine Science and Technology, Qingdao, China; ^4^ Center for Ocean Mega-Science, Chinese Academy of Sciences, Qingdao, China; ^5^ The Innovation of Seed Design, Chinese Academy of Sciences, Wuhan, China

**Keywords:** gene expression, disease resistance, molecular marker, *Vibrio parahaemolyticus*, *Litopenaeus vannamei*

## Abstract

Acute hepatopancreatic necrosis disease (AHPND) has caused a heavy loss to shrimp aquaculture since its outbreak. *Vibrio parahaemolyticus* (VP_AHPND_) is regarded as one of the main pathogens that caused AHPND in the Pacific white shrimp *Litopenaeus vannamei*. In order to learn more about the mechanism of resistance to AHPND, the resistant and susceptible shrimp families were obtained through genetic breeding, and comparative transcriptome approach was used to analyze the gene expression patterns between resistant and susceptible families. A total of 95 families were subjected to VP_AHPND_ challenge test, and significant variations in the resistance of these families were observed. Three pairs of resistant and susceptible families were selected for transcriptome sequencing. A total of 489 differentially expressed genes (DEGs) that presented in at least two pairwise comparisons were screened, including 196 DEGs highly expressed in the susceptible families and 293 DEGs in the resistant families. Among these DEGs, 16 genes demonstrated significant difference in all three pairwise comparisons. Gene set enrichment analysis (GSEA) of all 27,331 expressed genes indicated that some energy metabolism processes were enriched in the resistant families, while signal transduction and immune system were enriched in the susceptible families. A total of 32 DEGs were further confirmed in the offspring of the detected families, among which 19 genes were successfully verified. The identified genes in this study will be useful for clarifying the genetic mechanism of shrimp resistance against *Vibrio* and will further provide molecular markers for evaluating the disease resistance of shrimp in the breeding program.

## Introduction

*Litopenaeus vannamei* is a commercially important aquaculture species, making up about 85% of total shrimp production in China (Qin et al., 2018). However, the shrimp aquaculture industry is continuously affected by the outbreak of viral and bacterial diseases, which have caused mass mortality and considerable economic losses. *Vibrio parahaemolyticus* carrying the PirA and PirB toxin genes in its plasmid (VP_AHPND_) is one of the most destructive pathogens in shrimp aquaculture, causing acute hepatopancreatic necrosis disease (AHPND) or early mortality syndrome (EMS) in *L. vannamei* (Lee et al., 2015). VP_AHPND_ also caused AHPND in *Penaeus monodon* and *Exopalaemon carinicauda* (Soonthornchai et al., 2016; Ge et al., 2018). Therefore, prevention and control of AHPND are urgently needed in shrimp aquaculture.

Genetic selective breeding of disease resistance broodstock is a feasible and sustainable approach for the disease control. It has been proved to be efficient in controlling Taura syndrome virus (TSV). A disease-resistant line of *L. vannamei* against TSV had been established, and 18.4% increase in survival rate against TSV infection was obtained after one generation selection (Argue et al., 2002). For the selection of White spot syndrome virus (WSSV) resistance in *L. vannamei*, it showed that the average survival rates of generations G2 to G5 were 5.57%, 7.78%, 9.52%, and 13.79%, respectively (Huang et al., 2012). After three successive generation selection, the survival rates of *E. carinicauda* to VP_AHPND_ increased from 26.67% to 36.67% (Ge et al., 2018).

With the development of molecular biology, genomics approach offers a new possibility for accelerating the genetic selection process (Zhang X. et al., 2019). Identification of the major genes associated with disease resistance is the first step for marker-assisted selection (MAS) or gene-assisted selection (GAS) (Arora et al., 2019). So far, several genes associated with disease resistance have been reported in aquatic animals. Polymorphism of *LvALF* and *TRAF6* was reported to be associated with the resistance to WSSV in shrimp (Wang et al., 2011; Liu et al., 2014b; Liu et al., 2014a; Yu et al., 2017; Zhang Q. et al., 2019). A major quantitative trait locus (QTL) for the resistance of Atlantic salmon (*Salmo salar*) against infectious pancreatic necrosis (IPN) was discovered, which has been already applied in marker-assisted breeding of IPN-resistant fish (Houston et al., 2008; Moen et al., 2009; Woldemariam et al., 2020). However, knowledge about the resistance to AHPND in shrimp is still poorly understood. A comparison of individuals or families with significant phenotype difference by transcriptome sequencing is an efficient way for screening the trait-associated genes. Based on the transcriptome data, myosin, myosin heavy chain, and chitinase were proved to be related to growth performance in *L. vannamei* (Santos et al., 2018). Transcriptome comparison between the families with high growth rate and low growth rate also illustrated that the genes related to cuticle, chitin, and muscle proteins were upregulated exclusively in higher growth families (Santos et al., 2021). Besides, the gene profiles of the *Vibrio*-resistant and *Vibrio*-susceptible *Meretrix petechialis* families were analyzed, and several genes such as *Big-Def*, *CTL9*, and *Bax* were identified as candidate resistance-associated genes (Jiang et al., 2017).

In our previous work, we have carried out systematic family selection for the resistance trait to VP_AHPND_ in *L. vannamei*. A total of 95 families were subjected to VP_AHPND_ challenge test, and the results showed that significant resistance variations existed between different families. In the present study, we selected three AHPND resistant and three susceptible families for transcriptome sequencing and explored the differentially expressed genes (DEGs) in resistant and susceptible families. Several genes related to the resistance of shrimp against AHPND were identified. These genes might be developed as effective molecular markers for evaluating the disease resistance of shrimp, which could facilitate molecular marker-assisted breeding of shrimp.

## Materials and Methods

### Selection Resistant and Susceptible Families Against Acute Hepatopancreatic Necrosis Disease

**FIGURE 1:**
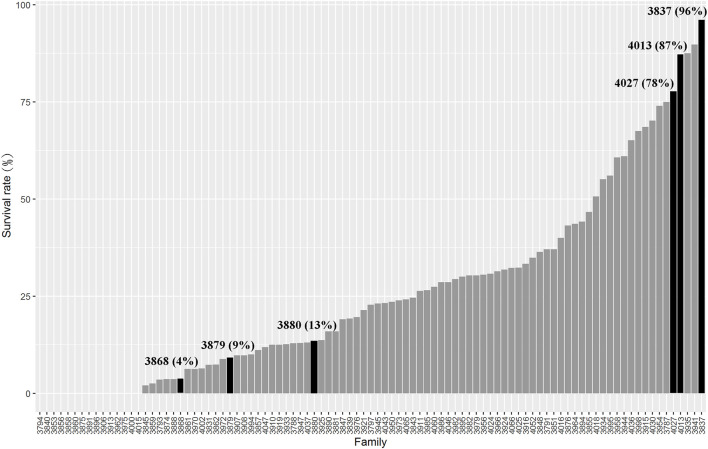
Survival rate of different families challenged by VP_AHPND_.

The full-sib families of *L. vannamei* were produced and stocked separately in Hainan Grand Suntop Ocean Breeding Co., Ltd, Wenchang, China. In order to identify the resistance of AHPND, the shrimp families were challenged by VP_AHPND_ each year, and the families with high survival rate were mated to generate the next-generation families. In 2018, 95 full-sib families with an average body weight of 2.10 g were selected for evaluation of resistance. For the challenge experiment, VP_AHPND_ was prepared according to the method described by Zhang Q. et al. (2019). About 100 healthy shrimp from each family were subjected to VP_AHPND_ immersion infection. Before the experiment, the shrimp were kept in the aquarium at a temperature of 26°C ± 1°C for 3 days to acclimate them to the culture conditions. Then, the VP_AHPND_ were added to the aquarium to make the concentration of VP_AHPND_ as 5 × 10^6^ CFU/ml. Then, the dead shrimp were checked every 2 h. The mortality of each family was recorded for 72 h. The survival rates of the tested families are presented in Figure 1.

Considering the survival rate, growth stage, and pedigree information, three resistant families (VR4013, VR3837, and VR4027) and three susceptible families (VS3868, VS3879, and VS3880) were selected for transcriptome sequencing. The survival rates of three resistant families VR4013 (1.96 ± 0.21 g), VR3837 (2.14 ± 0.33 g), and VR4027 (2.21 ± 0.26 g) were 87%, 96%, and 78%, and those of three susceptible families VS3868 (1.87 ± 0.24 g), VS3879 (2.09 ± 0.28 g), VS3880 (2.19 ± 0.30 g) were 4%, 9%, and 13%, respectively (Figure 1). Based on their pedigree information, the families VR4013 and VS3868 were genetically related; therefore, the DEGs were analyzed between VR4013 and VS3868 in order to avoid the effects of genetic difference as far as possible. According to information of the growth stage and pedigree information, DEGs were analyzed between VR3837 and VS3879, and VR4027 and VS3880, respectively.

For each family, the cephalothoraxes of nine individuals were collected, and three individuals were mixed together as one sample, so each family contained three samples. All cephalothorax samples of the six families were rapidly frozen in liquid nitrogen and stored at −80°C for further transcriptome sequencing.

### Total RNA Extraction, Library Construction, and Sequencing

Total RNA of the cephalothorax was extracted using RNAiso Plus (Takara, Japan) according to the manufacturer’s instructions. RNA purity and concentration were evaluated by electrophoresis on 1% agarose gel and quantified by NanoDrop 2000 spectrophotometer (Thermo Fisher Scientific, Waltham, MA, USA). Subsequently, mRNA was enriched by Oligo (dT) beads. Then the enriched mRNA was fragmented into short fragments using fragmentation buffer and reverse-transcribed into the first-strand cDNA with random primers. Second-strand cDNA was synthesized by DNA polymerase I, RNase H, dNTP, and buffer. Then the cDNA fragments were purified with QiaQuick PCR extraction kit, end repaired, poly (A) added, and ligated to Illumina sequencing adapters. The suitable fragments were selected by agarose gel electrophoresis. After PCR amplification, the libraries were sequenced using Illumina HiSeqTM 2,500 by Genedenovo Biotechnology Co., Ltd (Guangzhou, China).

### Bioinformatics Analysis

Clean reads were obtained by removing reads containing adapters or more than 10% of unknown nucleotides (N), low-quality reads, and rRNA from the raw data. Then the reads of each sample were mapped to reference genome (Zhang X. et al., 2019) by TopHat2 (version 2.0.3.12) (Kim et al., 2013). The reconstruction of transcripts was carried out with software Cufflinks (Trapnell et al., 2012). All reconstructed transcripts were aligned to reference genome, and novel genes were aligned to databases including National Center for Biotechnology Information (NCBI) nonredundant (Nr) database, Swiss-Prot database, and Kyoto Encyclopedia of Genes and Genomes (KEGG) database to obtain protein functional annotation. DEGs between susceptible and resistant libraries were analyzed using the edgeR package (http://www.rproject.org/), with a fold change ≥2 and a false discovery rate (FDR) <0.05. DEGs were then subjected to enrichment analysis of Gene Ontology (GO) functions and KEGG pathways, which were performed using the OmicShare tools (http://www.omicshare.com/tools).

### Gene Set Enrichment Analysis

As a complement to the differential expression analyses, gene set enrichment analysis (GSEA) for all 27,331 expressed genes was performed by GSEA software v4.10 (https://www.gsea-msigdb.org/gsea/downloads.jsp) (Subramanian et al., 2005). The submitted gene list was ranked by gene expression value using Signal2Noise method. GSEA was performed with default algorithm as 1,000 permutations. GO gene sets (10,192 gene sets) and KEGG subset of canonical pathways (186 gene sets) were used as enrichment input, which were from Molecular Signatures database (MSigDB, https://www.gsea-msigdb.org/gsea/msigdb/collections.jsp). Nominal *p*-value < 0.05 and FDR < 0.25 were considered as statistically significant.

### Evaluation on the Transcriptome Results by Real-Time Quantitative PCR

Six genes were selected from each comparison group to evaluate the transcriptome sequencing result by RT-qPCR. About 1 mg of total RNA was used to synthesize cDNA by PrimeScript™ RT reagent Kit with gDNA Eraser kit (Takara, Japan) according to the manufacturer’s instructions. Gene-specific primers (Table 1) were designed using Primer 5, where 18S rRNA was used as the reference gene for RT-qPCR analysis. RT-qPCR was conducted with the following conditions: denaturation at 94°C for 2 min, 40 cycles of 94°C for 30 s, annealing temperature for 20 s, and 72°C for 30 s. Each sample had four technical replicates. The specificity of the primer set was checked by melting curve analysis. The relative expression level was calculated with 2^−∆∆Ct^ method (Livak and Schmittgen, 2001).

**TABLE 1:** Primer sequences and annealing temperature used for RT-qPCR.

Gene name	Forward primer (5′–3′)	Reverse primer (5′ to 3′)	Product size (bp)	Tm (°C)
ncbi_113822350	ATT​CCC​TAC​AGT​GAC​GAC​TA	GCC​AAA​AGA​TTC​TCT​CAT​GC	132	51
ncbi_113828004	GTT​CCA​AAC​CCT​ACT​TGT​CT	CTA​TGT​CCA​AAA​CGG​AAT​GC	106	51
XLOC_016514	AGA​TGC​TTC​CCT​GGA​TCA​ACC	TGG​ACT​CTC​CAT​TCC​GAT​GTT​C	127	57
XLOC_016534	TCG​CCA​TGA​AGA​ACT​GGT​CA	GCA​AAT​TGA​AGG​CGT​CAG​CA	96	56
ncbi_113824827	TTG​CGA​GAC​AGA​CCA​ACC​AG	CAG​GTG​CAA​TCT​TCA​TCG​CC	133	55
ncbi_113816316	TTCCTCCCGCAAGACAAG	GAGGGAGGGTTGGGTTTT	150	55
ncbi_113816839	CTT​TCG​GGA​GGG​AGC​GTA​T	ACG​GGA​ATA​GTC​CAT​CCA​AGT	162	56
ncbi_113810874	ACC​CGC​TGT​CCG​CTC​TAC​CA	TGT​CCC​AGC​CGC​AGC​TCA​AC	118	64
ncbi_113805286	GGGCAACTTACGGCTTCT	TTCGTGCCAATGGGTTTC	131	54
ncbi_113826200	ATTGCAGCACCGTCTCCT	TCCCTCAGGCAGACTTCG	91	57
XLOC_026751	TCTTGTGCCTCGCTGTGG	GGT​GAT​GTG​CGT​GAT​CTT​CTT	149	57
ncbi_113826199	CTCACCGCTGCGAGGATT	TCCCTCAGGCAGACTTCG	106	58
XLOC_023290	TCTGCTGGTGATGATGGT	GTCATCGGGAGAACAACT	142	52
ncbi_113808761	CCG​CAA​TGC​TGT​AGA​AGG​AC	CGG​CGG​TCA​GAG​TGG​AGA​T	149	58
ncbi_113802520	CTTCTTGCCGTGTTTGCC	ACG​ATG​CCG​TCT​CCT​GTC​T	164	57
ncbi_113815780	ACT​CAT​AAC​CCA​CCG​CCA​CT	TCGTCAGGGACCCAGCAA	154	59
ncbi_1,13820830	AAG​CCG​AAC​TTG​GAG​GAC​C	CGG​ATG​AAC​TTA​CCG​AAA​CG	110	56
XLOC_016349	CATCAAGCCCAAACCACC	TCT​TCT​CCA​GCC​AGC​CAC​T	104	58
XLOC_016348	ATT​GGA​CGC​AAG​GAG​TAT​GG	CCTGGGCTGGTTGATGAG	146	55
ncbi_1,13817858	GAG​GAT​GGG​CTG​AAA​TGT​G	GTC​CAG​CAA​CTC​TGA​AGT​ATG​A	155	54
ncbi_113825958	GGA​ACA​GCA​GAC​GGG​AGT​G	CAACGAAGCATTGGTGGC	93	57
ncbi_113828431	CCG​TCA​CCA​ACA​CCC​ATA​A	AGCAGCCACCCAAGGAAA	150	56
ncbi_113807930	GCTCGTCACCACAACCAT	CGAAGATGGGAGGCAGGT	145	56
ncbi_113816695	GAG​CAC​CTC​GCT​TTC​TGT​TT	CAT​GAC​TTG​GGT​TCA​GGT​TTA	107	54
ncbi_113804592	TCA​CGG​AGT​GCC​GCT​ACG​AT	TCC​CTG​TTG​CGG​ATG​TCC​TG	187	60
ncbi_113813557	CTG​CCA​GTG​GAA​CAC​GCT​AT	GCG​GTG​CTA​GGA​ACG​TAA​CTA​A	186	57
ncbi_1,13817635	ATGCTGACAAGGCGAATA	AAGAGTCAGACCCGCAAG	159	52
ncbi_113830625	CGTGAATCGCAGTCCCTA	GTGGTCGCTTCCTCTTCC	100	55
ncbi_113807689	GGCAGCCGCATCTTCATC	AGG​GCG​AAG​CGG​CGG​TTG​TT	176	60
ncbi_1,13819349	GTT​CCA​TAC​CGC​CGT​TAC​CA	CGA​GCA​ATT​TCG​CTT​ACA​ACA​CTA	119	55
ncbi_113806536	GCA​CTT​CCA​AAG​CCA​ACG​A	GAT​CTC​CTC​GGA​GTT​GTA​GCG	119	57
ncbi_113802817	CGTCGCTGGGCACAAGTA	AGCCGAAGTGTCCCGTTA	167	57
ncbi_1,13817262	AATGAGGCGGAGGAGCAG	CCT​TCC​AGG​TGG​CAG​ACA​G	92	58
ncbi_113821874	TAAGAAGGTCCAGAGGCG	AACCCACAAGGCCATACA	125	55
ncbi_113810465	CGC​TGG​TGG​GTG​TCG​TGA​T	CCGCTTGGCTGCTGAGAT	117	55
ncbi_113811111	CCGAGGTCAACTACGAGG	ACG​GGA​CTT​GGT​GGC​TGG​T	106	55
ncbi_113816327	AAACCCAACCCTCCCTCT	TCCTCCGTCTCCAACACC	136	54
ncbi_113821801	AAGGGCGTGGAAGGAATG	CTT​CAT​CTC​CTC​CTT​CTC​CTT​C	187	56
18S	TAT​ACG​CTA​GTG​GAG​CTG​GAA	GGG​GAG​GTA​GTG​ACG​AAA​AAT	136	56

### Validation of Candidate Genes in Descendant Families

In order to validate the identified DEGs, the descendant families of VS3868 and VR4013 were collected, and the DEGs were further validated in the descendant families by RT-qPCR. Family 4419 was produced by full-sib mating of family VS3868, and family 4253 was produced by full-sib mating of family VR4013. After being challenged by VP_AHPND_, the survival rate of family 4253 was over 3.5 times higher than that of family 4419 (60 vs. 17%). A total of 32 DEGs including the 16 DEGs shared in three pairwise comparisons and other 16 DEGs shared in two pairwise comparisons were selected, and they were verified in family 4419 and 4253. The primers designed by Primer 5 are listed in Table 1. The RT-qPCR was performed as described in *Evaluation on the Transcriptome Results by Real-Time Quantitative PCR*. A statistically significant difference was indicated with * (*p* < 0.05) and ** (*p* < 0.01) determined by *t*-test (SPSS Inc., Armonk, NY, USA).

## Results

### Transcriptome Sequencing Data

An overview of sequencing and assembly of the *L. vannamei* transcriptome is shown in Table 2. A total of 823,161,050 raw reads were obtained, in which 416,197,128 reads are for the resistant families and 406,963,922 reads for the susceptible families. The raw sequencing data were uploaded to the NCBI with the accession numbers SRR15533118–SRR15533135. After low-quality reads were filtered out, a total of 96.55% and 96.85% reads were retained for the resistant and susceptible groups, respectively. After the reads were mapped to the reference genome, a total of 27,331 annotated genes were obtained, of which 2,344 (9.86%) were newly annotated genes.

**TABLE 2:** Summary of transcriptome sequencing and assembly of the transcriptome from *Litopenaeus vannamei*.

Sample	Raw reads	Clean reads	Percentage remained (%)	Gene number
VR4013-1	43,410,372	42,125,030	97.04	20,485
VR4013-2	57,672,500	55,606,214	96.42	20,677
VR4013-3	51,479,718	49,787,374	96.71	21,014
VR3837-1	38,338,506	36,947,962	96.37	19,828
VR3837-2	37,704,754	36,467,784	96.72	19,592
VR3837-3	47,019,886	45,527,220	96.83	20,211
VR4027-1	56,229,492	54,118,212	96.25	21,034
VR4027-2	39,363,752	37,908,938	96.30	20,832
VR4027-3	44,978,148	43,327,096	96.33	20,310
VS3868-1	42,426,736	41,120,350	96.92	20,912
VS3868-2	42,975,552	41,754,004	97.16	20,621
VS3868-3	41,490,144	40,033,320	96.49	20,573
VS3880-1	51,220,018	49,705,886	97.04	20,157
VS3880-2	45,819,944	44,689,240	97.53	20,289
VS3880-3	42,021,826	40,653,124	96.74	20,005
VS3879-1	49,914,612	48,181,284	96.53	20,111
VS3879-2	43,730,322	42,164,296	96.42	19,971
VS3879-3	47,364,768	45,863,282	96.83	20,366

### Identification of Differentially Expressed Genes Between Resistant and Susceptible Families

**FIGURE 2:**
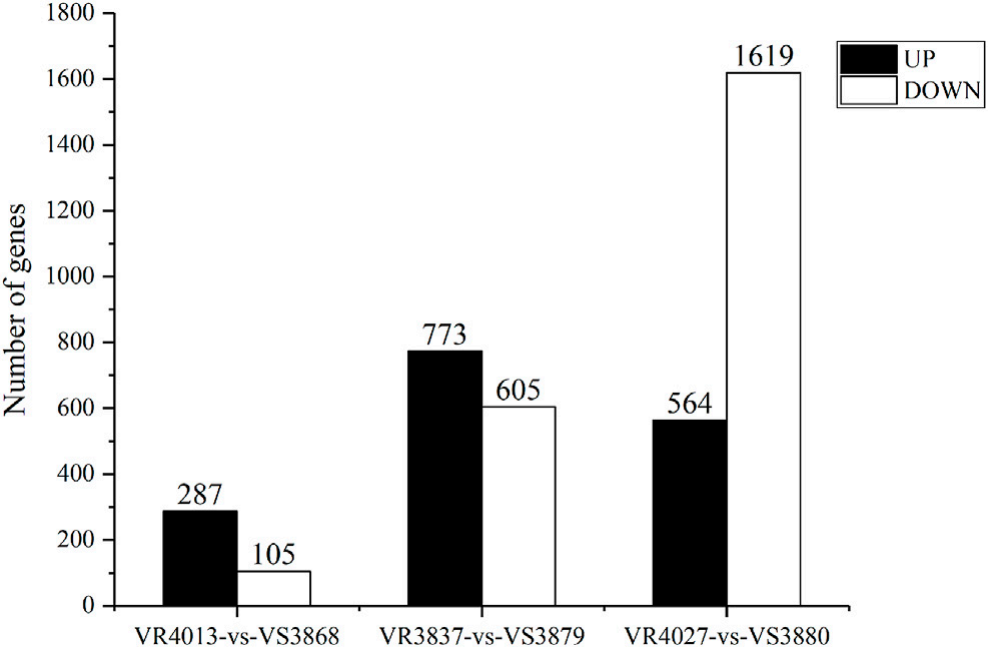
The amount of differentially expressed genes (DEGs) between resistant and susceptible families. UP represents highly expressed genes in susceptible families. DOWN represents highly expressed genes in resistant families.

To identify DEGs involved in VP_AHPND_ resistance, we used FPKM value for comparing the expression levels between the resistant families and susceptible families. A total of 392 unigenes showed differential expression patterns between families VR4013 and VS3868, in which 287 unigenes were highly expressed in VS3868 and 105 unigenes were highly expressed in VR4013 (Figure 2). A total of 1,378 unigenes were differentially expressed between VR3837 and VS3879, including 773 unigenes highly expressed in VS3879 and 605 unigenes highly expressed in VR3837. A total of 2,183 unigenes were differentially expressed between VR4027 and VS3880, including 564 unigenes highly expressed in VS3880 and 1,619 unigenes highly expressed in VR4027. Six DEGs selected from each comparison group were validated by RT-qPCR. The results showed that all of them were consistent with the transcriptome data (Supplementary Figure S1).

**FIGURE 3:**
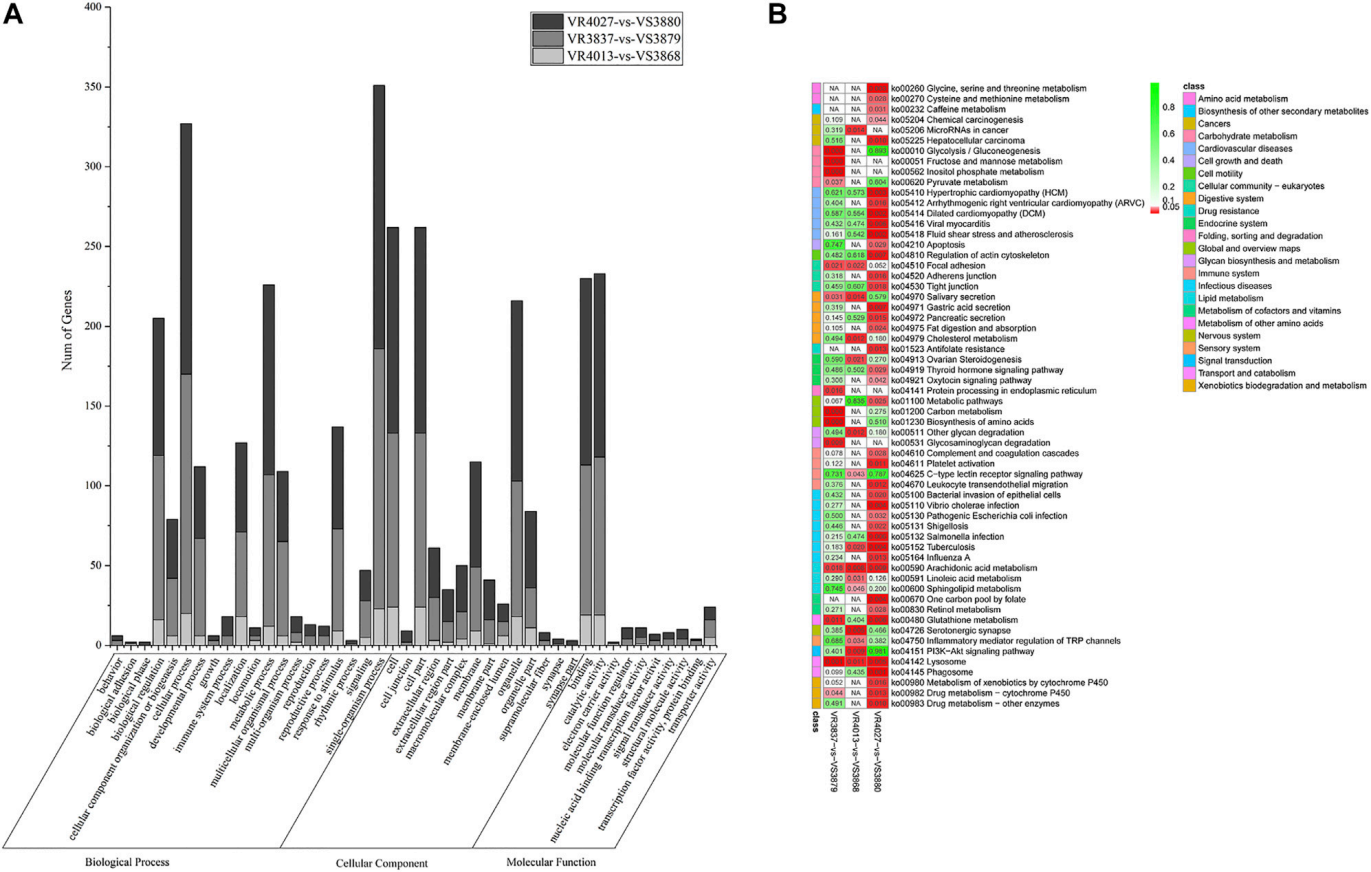
Enrichment analysis of differentially expressed genes (DEGs). **(A)** Gene Ontology (GO) terms distribution for the DEGs. Light gray represents VR4013-vs.-VR3868. Gray represents VR3837-vs.-VR3879. Dark gray represents VR4027-vs.-VR3880. The *x*-axis indicates the name of GO subcategories. The *y*-axis represents the number of genes. **(B)** Kyoto Encyclopedia of Genes and Genomes (KEGG) pathways enriched about DEGs in VR4013-vs.-VR3868, VR3837-vs.-VR3879, and VR4027-vs.-VR3880.

In order to analyze the function of DEGs between resistant and susceptible families, GO functional enrichment analysis was performed. Interestingly, we found that GO terms of DEGs were very similar among three pairs of families, VR4013-vs.-VS3868, VR3837-vs.-VS3879, and VR4027-vs.-VS3880, which is shown in Figure 3A. In the biological process category, single-organism process was the most enriched subclasses, followed by cellular process. In the cellular component category, cell and cell part were the two most enriched subclasses. While in the molecular function category, catalytic activity and binding were the two most enriched subclasses. KEGG pathway enrichment analysis of three pairwise comparisons is presented in Figure 3B. In VR4013-vs.-VS3868, “Serotonergic synapse,” “Arachidonic acid metabolism,” and “PI3K–Akt signaling pathway” were the three most enriched pathways, and “Arachidonic acid metabolism” was also included in top 20 of enrichment pathways in VR3837-vs.-VS3879 and VR4027-vs.-VS3880 (*p* < 0.05).

### Differentially Expressed Genes Shared in More Than Two Pairwise Comparisons

**FIGURE 4:**
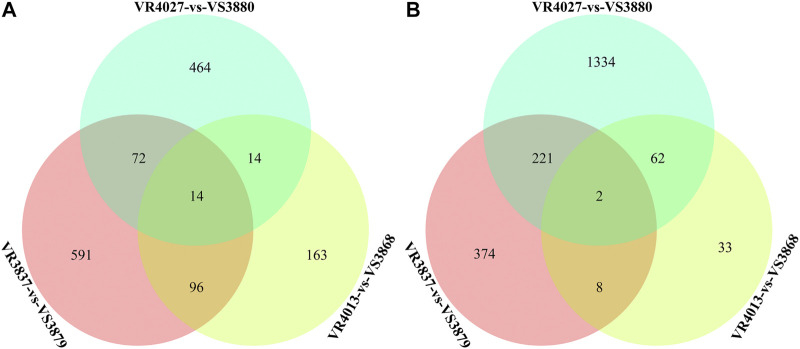
Venn diagrams for three pairwise comparisons of resistant and susceptible families. **(A)** Differentially expressed genes (DEGs) with high expression in susceptible families. **(B)** DEGs with high expression in resistant families.

Venn diagrams for DEGs of the three pairwise comparisons are shown in Figure 4. A total of 489 DEGs were shared in at least two comparisons, including 196 DEGs highly expressed in the susceptible families and 293 DEGs highly expressed in resistant families (Supplementary Table S1). A total of 16 DEGs were shared in three pairwise comparisons, among which the trophoblast glycoprotein, SE-cephalotoxin-like, peroxidasin-like protein, phosphoenolpyruvate carboxykinase, cytochrome P450, and serpin B6-like isoform X2 genes were identified (Table 3). In order to reduce the false positives, we considered these 489 DEGs as the candidate genes associated with the resistance of shrimp against to VP_AHPND_.

**TABLE 3:** Gene name, gene annotation, and the verification in hepatopancreas (Hep) and stomach (St) of 16 DEGs that were shared in three pairwise comparisons.

Gene name	Gene annotation	Verification (*p-*value)	
		Hep	St
ncbi_113805286	NA^a^		√(0.001)
ncbi_113810874	Trophoblast glycoprotein		√(0.024)
ncbi_113826200	NA	√(0.025)	
XLOC_016514	NA		√(0.004)
XLOC_016534	NA		
ncbi_113822350	SE-cephalotoxin-like	√(0.004)	√(0.01)
ncbi_113824827	Prolow-density lipoprotein receptor-related protein 1-like	√(0.018)	√(0.013)
ncbi_113828004	Peroxidasin-like protein		√(0.02)
ncbi_113819349	Phosphoenolpyruvate carboxykinase, cytosolic		
ncbi_113807689	Cytochrome P450		
XLOC_026751	NA	√(0.006)	√(0.014)
ncbi_113826199	Single insulin-like growth factor-binding domain protein-2	√(0.003)	√(0.009)
XLOC_023290	NA	√(0.012)	
ncbi_113825958	Serpin B6-like isoform X2		
ncbi_113816327	NA		
ncbi_113816316	NA	√(0.023)	

Note. The threshold for significance is *p*-value < 0.05.

DEGs, differentially expressed genes.

a NA indicates that the function of gene is unknown.

**FIGURE 5:**
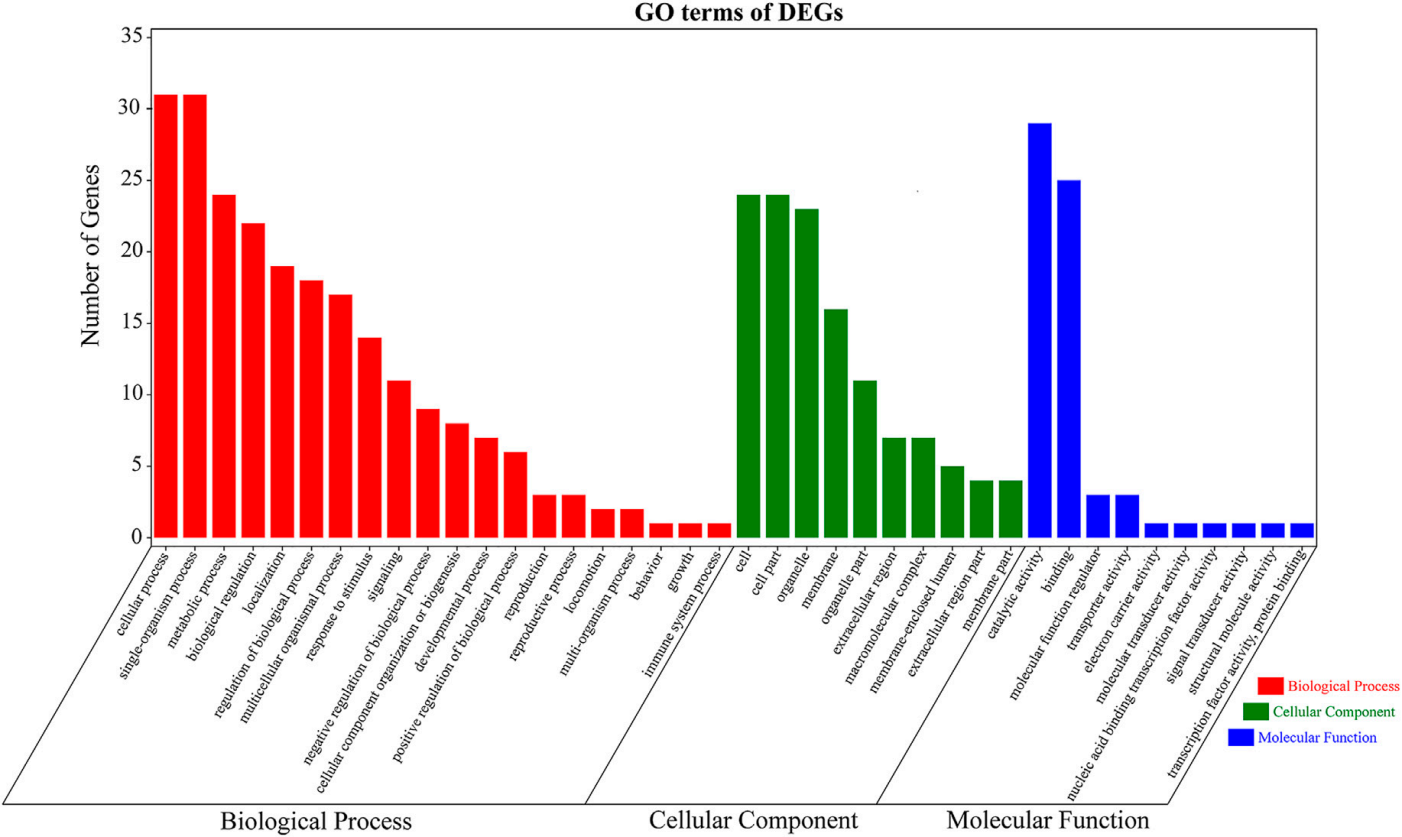
Gene Ontology (GO) terms of 489 differentially expressed genes (DEGs) for the transcriptomes of *Litopenaeus vannamei*. The *x*-axis indicates the name of GO subcategories. The *y*-axis indicates the number of genes. Red indicates biological process. Green indicates cellular component. Blue displays molecular function.

These 489 DEGs were involved in various GO classifications. For the biological process-related genes, most were involved in “single-organism process,” “cellular process,” and “metabolic process.” Most of the cellular component-related genes were associated with “cell,” “cell part,” and “organelle.” And “catalytic activity” and “binding” in the molecular function ontology were the major enriched terms (Figure 5). The results were consistent with GO functional enrichment analysis in Figure 3A.

### Differentially Expressed Genes with High Expression in Resistant Families

There were 293 DEGs highly expressed in resistant families. A total of nine genes annotated as myosin were highly expressed, and the other genes were involved in energy metabolism such as trypsin, chymotrypsinogen A (ChyA), pancreatic lipase (PL), serine protease (SP), aminopeptidase, and phospholipase (Table 4). There were two genes encoding glyceraldehyde 3-phosphate dehydrogenase (GAPDH) and triosephosphate isomerase (TPI), which were involved in the glycolysis pathway (Eanes, 2011).

**TABLE 4:** Myosin- and energy metabolism-related DEGs with high expression in VP_AHPND_-resistant *Litopenaeus vannamei* families.

Gene ID	Description	Species
ncbi_113815780	Myosin-16-like	*Lepisosteus oculatus*
ncbi_113820830	Myosin-16 isoform X2	*L. oculatus*
XLOC_031566	Myosin heavy chain, muscle-like isoform X7	*Hyalella azteca*
ncbi_113816531	Myosin-7	*Chelonia mydas*
XLOC_016349	Myosin heavy chain type 2	*L. vannamei*
XLOC_016348	Myosin heavy chain type 2	*Penaeus monodon*
XLOC_016355	Myosin heavy chain type b	*Marsupenaeus japonicus*
XLOC_015656	Myosin heavy chain type b	*M. japonicus*
ncbi_113802000	Myosin heavy chain, partial	*Rana catesbeiana*
XLOC_009141	Actin 2	*Nilaparvata lugens*
ncbi_113815891	Tryptase-like	*Lates calcarifer*
ncbi_113822347	Tryptase-2-like	*Scleropages formosus*
ncbi_113813760	Trypsin-like	*Sinocyclocheilus rhinocerous*
ncbi_113809217	Trypsin-2-like	*S. rhinocerous*
ncbi_113814573	Chymotrypsinogen A	*Ovis aries musimon*
ncbi_113806996	Serine protease 33-like	*Python bivittatus*
ncbi_113800311	Serine protease 27-like	*L. calcarifer*
XLOC_005102	Aminopeptidase N-like	*H. azteca*
ncbi_113815289	Aminopeptidase N	*Ornithorhynchus anatinus*
ncbi_113815282	Aminopeptidase N	*Octodon degus*
ncbi_113815313	Aminopeptidase N	*Ochotona princeps*
ncbi_113815275	Aminopeptidase Ey-like	*Xenopus laevis*
ncbi_113811393	Aminopeptidase Ey-like	*X. laevis*
ncbi_113804819	Aminopeptidase Ey-like	*X. laevis*
ncbi_113804804	Aminopeptidase N, partial	*Chlorocebus sabaeus*
XLOC_009027	Cathepsin l, partial	*L. vannamei*
ncbi_113807506	Pancreatic lipase-related protein 2-like	*Chaetura pelagica*
ncbi_113821709	Phospholipase A2	*Pagrus major*
ncbi_113808761	Alkaline phosphatase, tissue-nonspecific isozyme-like	*S. rhinocerous*
ncbi_1,13820123	Glyceraldehyde 3-phosphate dehydrogenase	*Esox lucius*
ncbi_113802551	Triosephosphate isomerase A	*Astyanax mexicanus*

Note. DEGs, differentially expressed genes.

### Differentially Expressed Genes with High Expression in Susceptible Families

A total of 196 DEGs highly expressed in the susceptible families were discovered. According to annotation and function, some immune-related genes were observed to be upregulated in the susceptible families (Table 5). A total of five genes related to the prophenoloxidase (proPO) system, including C-type lectin, SP, and SP inhibitors (serpin and *α*-2-macroglobulin) showed high expression in the susceptible families. Several genes are related to metabolic process, including DBH-like monooxygenase protein 1, cytochrome b5, and cytochrome P450. Moreover, six genes annotated as *β*-arrestin were also highly expressed in the VP_AHPND_-susceptible families.

**TABLE 5:** Immunity-related DEGs with high expression in VP_AHPND_-susceptible *Litopenaeus vannamei* families.

Gene ID	Description	Species
ncbi_113817858	C-type lectin domain family 4 member E-like isoform X3	*Lates calcarifer*
ncbi_113825958	Serpin B6-like isoform X2	*Sarcophilus harrisii*
ncbi_113828431	Serine protease 42-like	*Chrysochloris asiatica*
ncbi_113808061	*α*-2-Macroglobulin-like protein 1	*Pseudopodoces humilis*
ncbi_113808062	*α*-2-Macroglobulin-like protein 1	*Coturnix japonica*
ncbi_113816695	DBH-like monooxygenase protein 1	*Otolemur garnettii*
ncbi_113804592	DBH-like monooxygenase protein 1	*O. garnettii*
ncbi_113813557	Cytochrome b5-like	*Salmo salar*
ncbi_113807689	Cytochrome P450	*Danio rerio*
ncbi_113829525	*β*-Arrestin-2 isoform X2	*Heterocephalus glaber*
ncbi_113826122	*β*-Arrestin-2	*Pelodiscus sinensis*
ncbi_113815019	*β*-Arrestin-2	*Lepisosteus oculatus*
ncbi_113822594	*β*-Arrestin-1-like, partial	*Takifugu rubripes*
ncbi_113804603	*β*-Arrestin-1, partial	*Kryptolebias marmoratus*
ncbi_113807566	*β*-Arrestin-1	*Gekko japonicus*

Note. DEGs, differentially expressed genes.

### Gene Set Enrichment Analysis

**FIGURE 6:**
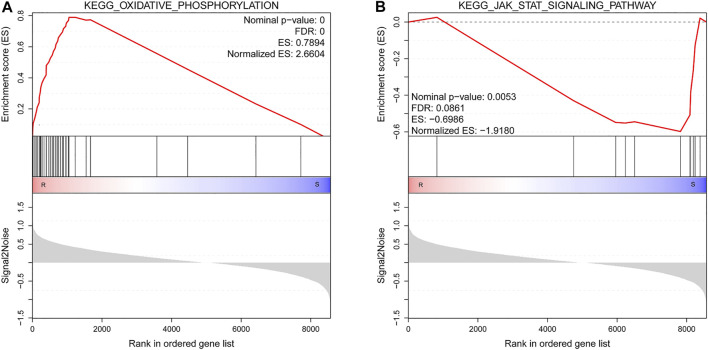
Gene set enrichment analysis (GSEA) of Kyoto Encyclopedia of Genes and Genomes (KEGG) subset of canonical pathways. **(A)** The most significantly enriched pathway in resistant families. **(B)** The most significantly enriched pathway in susceptible families. R, resistant samples; S, susceptible samples. Nominal *p*-value, false discovery rate (FDR), enrichment score (ES), and normalized ES were determined by the GSEA software and were indicated within each enrichment plot.

By GSEA of GO gene set, there were a total of 265 significantly enriched gene sets between resistant and susceptible groups (nominal *p*-value < 0.05 and FDR < 0.25). The top 10 gene sets enriched in resistant families were all involved in energy metabolism process, while most gene sets enriched in the susceptible families were related to signal transduction and immunity, such as G protein-coupled receptor activity, G protein-coupled receptor signaling pathway, and pattern recognition receptor signaling pathway (Table 6). Figure 6 shows GSEA of KEGG subset of canonical pathways. The most significantly enriched pathway in resistant families was oxidative phosphorylation (Figure 6A), which was also related to energy metabolism, supporting the results of GO gene sets. The most significantly enriched pathway in the susceptible families was JAK/STAT signaling pathway (Figure 6B), including protein inhibitor of activated STAT, cytokine-inducible SH2-containing protein, and tyrosine-protein phosphatase non-receptor type 11 isoform X2.

**TABLE 6:** GSEA of GO gene sets.

EP	Gene set	Size	NES	Nom *p*-val	FDR
R	Mitochondrial_protein_complex	78	2.70	0	0
R	Inner_mitochondrial_membrane_protein_complex	36	2.64	0	0
R	Cellular_respiration	64	2.61	0	0
R	Oxidative_phosphorylation	44	2.58	0	0
R	Mitochondrial_respiratory_chain_complex_assembly	33	2.57	0	0
R	Respiratory_electron_transport_chain	40	2.54	0	0
R	Respiratory_chain_complex	27	2.53	0	0
R	ATP_synthesis_coupled_electron_transport	35	2.52	0	0
R	NADH_dehydrogenase_complex_assembly	26	2.49	0	0
R	Respirasome	35	2.44	0	0
S	G_Protein_coupled_receptor_activity	52	−2.16	0	0.119
S	G_Protein_coupled_receptor_signaling_pathway	115	−2.11	0	0.130
S	Tight_junction	18	−2.10	0	0.101
S	Positive_regulation_of_blood_circulation	8	−2.10	0	0.078
S	Solute_sodium_symporter_activity	32	−2.10	0	0.066
S	Neurotransmitter_binding	17	−2.10	0	0.055
S	Neuromuscular_process_controlling_balance	10	−2.09	0	0.048
S	Apical_junction_complex	18	−2.05	0.003	0.072
S	Serine_type_endopeptidase_inhibitor_activity	17	−2.05	0.003	0.069
S	Pattern_recognition_receptor_signaling_pathway	27	−2.02	0	0.085

Note. EP, enrichment in phenotype, gene sets enriched in nine resistant (R) samples or nine susceptible (S) samples; Size, number of genes in the gene set; NES, normalized enrichment score; NOM *p*-value, nominal *p*-value, the statistical significance of the enrichment score; FDR, false discovery rate; GSEA, Gene set enrichment analysis; GO, Gene Ontology.

### Verification of Candidate Genes in the Descendant Families

**FIGURE 7:**
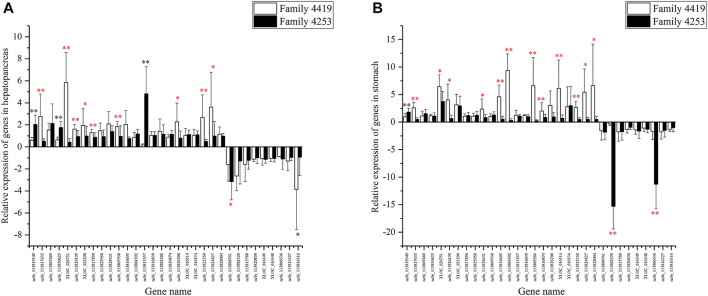
The relative expression of 32 differentially expressed genes (DEGs) in hepatopancreas **(A)** and stomach **(B)** from resistant family 4253 and susceptible family 4019. These data are expressed as the mean ± SD relative to the reference gene (18S rRNA). A statistically significant difference is indicated with * (*p* < 0.05) and ** (*p* < 0.01). Successfully verified genes are marked in red. Unsuccessfully verified genes are marked in black or without any mark.

A total of 32 genes were validated in descendant families. For the 16 DEGs shared by three pairwise comparisons, seven genes were verified successfully in the hepatopancreas, and eight genes were verified successfully in the stomach in their offspring (Table 3, *p* < 0.05). For the other 16 DEGs shared by two pairwise comparisons, three genes were successfully verified in hepatopancreas (*p* < 0.05) (Figure 7A), and six genes were verified successfully in the stomach (*p* < 0.05) (Figure 7B). In summary, a total of 19 genes were successfully verified in the hepatopancreas or stomach, including five genes verified successfully in two tissues.

## Discussions

It is generally considered that the VP_AHPND_ is an opportunistic pathogen. It is pathogenic to cultured shrimp at high concentration or under environmental deterioration condition (Hong et al., 2016). The physiology and health state of shrimp are closely related to disease resistance. It was estimated that the heritability of the resistance against VP_AHPND_ in *L. vannamei* was near-to-moderate, which indicated that the resistance of shrimp against VP_AHPND_ was hereditable (Wang et al., 2019). In this study, the resistant and susceptible families of *L. vannamei* were obtained through continuous challenge test, which provided reliable genetic material for analysis on the disease resistance of shrimp (Grimholt, et al., 2003; Wang et al., 2014). Understanding the gene expression character of disease-resistant families and the susceptible families could provide useful information for genetic dissection of disease resistance of shrimp.

Recently, an increasing number of transcriptome studies related to AHPND have been performed in *L. vannamei*, most of which focused on the genes involved in the immune response of shrimp during VP_AHPND_ infection (Qi et al., 2017; Maralit et al., 2018; Ng et al., 2018; Qin et al., 2018; Zheng et al., 2018). However, few works have focused on the correlation between the gene expression level and resistance phenotype of shrimp. In this study, we performed RNA-seq analyses to investigate the comparative expression profiles between resistant and susceptible families of shrimp. Gene expression profiles can underlie complex phenotype variations (Crawford and Powers, 1992). The gene expression variation is influenced by various genetic and environmental factors (Leder et al., 2014). Many studies proved that genetic factors influence gene expression in humans (Cheung and Spielman, 2002), mice (Sandberg et al., 2000), *Drosophila* (Jin et al., 2001), and yeast (Brem et al., 2002). To our knowledge, this is the first report about the comparison of the basal mRNA expression profiles from the omics level of AHPND-resistant and AHPND-susceptible families of *L. vannamei*. The gene expression levels influencing the phenotype variations could also be considered as molecular markers and could be used for genetic selection (Gilad et al., 2008). For aquaculture species, genes related to phenotype variations have been reported. The expression of BIRC7 was correlated with the survival in *Vibrio* challenge tests in clam *M. petechialis*, and gene expression variation of BIRC7 gene was heritable, indicating the feasibility of selective breeding by reliable genetically based markers (Jiang et al., 2018; Jiang et al., 2019).

In the present study, we found that DEGs highly expressed in the susceptible families of shrimp were mainly related to the immunity, which were in agreement with previous research results. It was reported that the basal expression levels of immune-related genes BIRC7 and NFIL3 were higher in *Vibrio*-susceptible clams (Jiang et al., 2018). In this study, several genes in proPO activation system showed a higher expression level in the susceptible families. A previous report showed that PPAE2 presented a higher expression level in the susceptible line of *L. vannamei* after AHPND challenge in comparison with the resistant line (Mai et al., 2021). This system is important for fighting against bacteria pathogens in penaeid shrimp (Amparyup, et al., 2013), and the increased activity of proPO system against *Vibrio* has been reported in *Fenneropenaeus indicus* (Sarathi et al., 2007), *L. vannamei* (Boonchuen et al., 2021), and *Macrobrachium rosenbergii* (Rao et al., 2015). The C-type lectin was also upregulated in the susceptible families of shrimp; the C-type lectin plays an important role in phagocytosis, melanization, respiratory burst, and coagulation; and it can also activate the proPO system (Junkunlo et al., 2012). Interestingly, C-type lectin 1-like and Crustin-P had significant higher expression levels in the AHPND-susceptible line of *L. vannamei* than resistant line at 24 h post-infection (Mai et al., 2021). In *Litopenaeus stylirostris*, the basal expression level of antimicrobial peptide was significantly higher in *Vibrio*-susceptible shrimp line (Lorgeril et al., 2008). In addition, *β*-arrestin also played a vital role in the antibacterial immunity of shrimp (Jiang et al., 2013; Sun et al., 2016). The GSEA also illustrated that partial immune-related genes were upregulated in the susceptible families; the JAK/STAT signaling pathway, which was an important signal transduction pathway regulating the immune response in invertebrates, was significantly enriched in the susceptible families (Yu et al., 2017).

Besides, the genes in immunity showed differential expression patterns between susceptible and resistant families; another major part of the DEGs was related to myosin and metabolism. In this study, myosin and many other genes related to metabolism were more active in resistant families. Myosin and actin play a diverse role in a wide range of functions such as cytoskeleton, muscle contraction, and immune response (Marston 2018; Sitbon et al., 2019). It was already reported that myosin light chain was related to the phagocytosis against invading pathogens in *Penaeus japonicus*, and the transcription level of myosin in WSSV-resistant shrimp was nearly two times higher than that in the control shrimp (Han et al., 2010). After pathogen infection, myosin and actin were significantly upregulated in shrimp (Shi et al., 2018; Ren et al., 2019). As for metabolism, we found that enzymes like trypsin, ChyA, PL, and SP and glycolysis pathway including GAPDH and TPI were highly expressed in the resistant families (Table 4). Meanwhile, the top 10 gene sets enriched in resistant families in the GSEA were all involved in energy metabolism process. The finding was consistent with the previous report that SP and ChyB had a significantly higher expression level in resistant shrimp line during the AHPND infection (Mai et al., 2021). Several studies have indicated that metabolic processes such as lipid metabolism and carbohydrate metabolic process in shrimp were greatly affected during AHPND infection (Velazquez-Lizarraga et al., 2019; Kumar et al., 2021). Taken together, the activated myosin, actin, and energy metabolism might indicate that the shrimp were healthier, which led to higher resistance of shrimp to disease.

After validation, several genes showed the same expression pattern in the offspring of susceptible and resistant families. These genes are possible to be developed as biomarkers for disease resistance of shrimp. It was already reported that gene expression profile could be used as an indicator for disease resistance trait. For example, Bsr-d1 RNA level in susceptible rice strain was twofold higher than that in resistant rice strain, and it has been further proved that inhibiting the expression of bsr-d1 could increase the rice resistance against blast infection (Li et al., 2017). In *E. carinicauda*, eight immune-related genes were suggested as potential disease-resistant parameters for evaluating the physiological status and disease-resistant capability of shrimp when infected with VP_AHPND_ (Ge et al., 2018). In this study, the 19 genes successfully verified in their descendant families were expected to be developed as biomarkers of shrimp resistance against *Vibrio*. Therefore, apart from sib challenge testing and molecular marker-assisted breeding, the gene expression level of these 19 genes could also be used as molecular markers for accelerating the breeding of disease-resistant varieties in *L. vannamei*.

In summary, this study integrated the VP_AHPND_-resistance phenotype variation and gene expression profiles to identify the genes related to disease resistance of shrimp. A total of 489 DEGs were identified between the resistant and susceptible families, and they were considered to be associated with the ability of VP_AHPND_ resistance in *L. vannamei*. Gene annotation and enrichment analysis revealed that the immune response and energy metabolism could influence resistance of shrimp against VP_AHPND_. The obtained data provide a fundamental basis for clarifying the genetic mechanism of resistance to bacterial pathogen, and the identified disease resistance genes of shrimp could accelerate the genetic breeding in shrimp aquaculture.

## Data Availability

The datasets presented in this study can be found in online repositories. The names of the repository/repositories and accession number(s) can be found in the article/Supplementary Material.
