# Supplementary figures and images for: Comparison of Gene Expression Between Resistant and Susceptible Families Against VPAHPND and Identification of Biomarkers Used for Resistance Evaluation in Litopenaeus vannamei
Source: Front Genet. 2021 Nov 26;12:772442. doi: 10.3389/fgene.2021.772442 (PMC8662381; doi:10.3389/fgene.2021.772442)

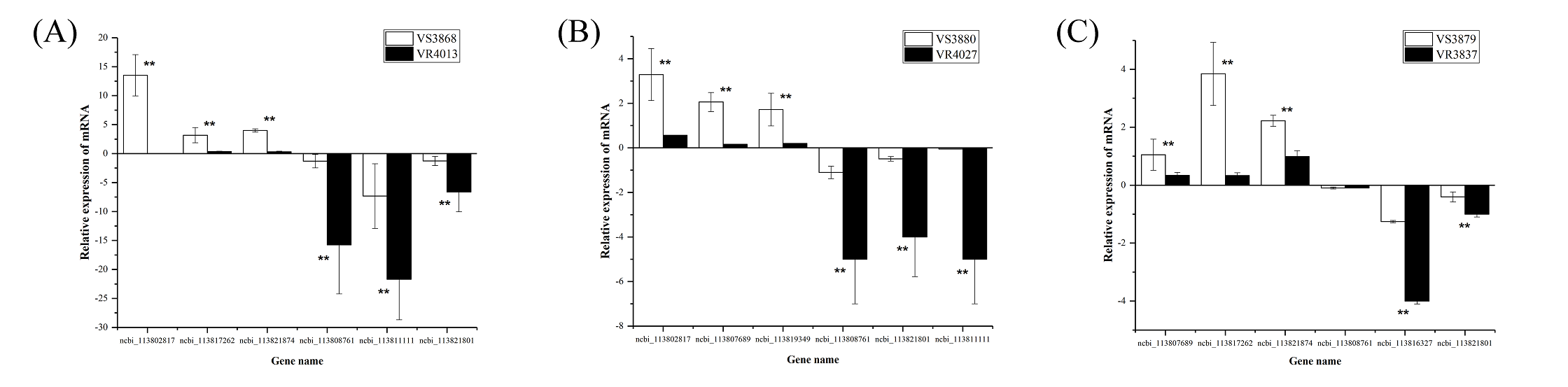

Supplement: Supplementary file 1 [file Image1.TIF]
